# AKT-mediated phosphorylation of Sox9 induces *Sox10* transcription in a murine model of HER2-positive breast cancer

**DOI:** 10.1186/s13058-021-01435-6

**Published:** 2021-05-13

**Authors:** Khalid N. Al-Zahrani, John Abou-Hamad, Julia Pascoal, Cédrik Labrèche, Brennan Garland, Luc A. Sabourin

**Affiliations:** 1 Centre for Cancer Therapeutics, Ottawa Hospital Research Institute, 501 Smyth Road, Ottawa, ON K1H 8L6 Canada; 2 Department of Cellular and Molecular Medicine, University of Ottawa, Ottawa, ON K1H 8M5 Canada

**Keywords:** Ste20-like kinase, Sox10, HER2-positive breast cancer, Sox9, AKT

## Abstract

**Background:**

Approximately 5–10% of HER2-positive breast cancers can be defined by low expression of the Ste20-like kinase, *SLK*, and high expression of *SOX10.* Our lab has observed that genetic deletion of SLK results in the induction of *Sox10* and significantly accelerates tumor initiation in a HER2-induced mammary tumor model. However, the mechanism responsible for the induction of *SOX10* gene expression in this context remains unknown.

**Methods:**

Using tumor-derived cell lines from MMTV-Neu mice lacking SLK and biochemical approaches, we have characterized the signaling mechanisms and relevant DNA elements driving *Sox10* expression.

**Results:**

Biochemical and genetic analyses of the *SOX10* regulatory region in SLK-deficient mammary tumor cells show that Sox10 expression is dependent on a novel −7kb enhancer that harbors three SoxE binding sites. ChIP analyses demonstrate that Sox9 is bound to those elements in vivo. Our data show that AKT can directly phosphorylate Sox9 in vitro at serine 181 and that AKT inhibition blocks Sox9 phosphorylation and Sox10 expression in SLK(-/-) tumor cells. AKT-mediated Sox9 phosphorylation increases its transcriptional activity on the Sox10 −7kb enhancer without altering its DNA-binding activity. Interestingly, analysis of murine and human mammary tumors reveals a direct correlation between the levels of active phospho-Sox9 S181 and Sox10 expression.

**Conclusions:**

Our results have identified a novel Sox10 enhancer and validated Sox9 as a direct target for AKT. As Sox10 is a biomarker for triple-negative breast cancers (TNBC), these findings might have major implications in the targeting and treatment of those cancers.

**Supplementary Information:**

The online version contains supplementary material available at 10.1186/s13058-021-01435-6.

## Background

Approximately, 12% of all new cancers yearly are reported as breast carcinomas. However, extensive heterogeneity and the complex biology and etiology for the various breast cancer subtypes make treatment difficult. Breast cancer subtypes are generally classified based on the expression of the estrogen receptor (ESR1), progesterone receptor (PGR), or the human epidermal growth factor receptor 2 (HER2) [1, 2]. The HER2-positive subtype, characterized by overexpression and amplification of HER2, accounts for 20–30% of all breast cancers and is associated with poor prognosis and aggressive cancers [3, 4]. The activation of the HER2 receptor through tyrosine phosphorylation [5, 6] results in the upregulation of proliferative and survival pathways [7, 8]. Although HER2 amplification is a critical event in the etiology of HER2+ breast cancers, the molecular mechanisms regulating its progression are not fully understood.

The Ste20-like kinase (SLK) has been shown to regulate multiple biological responses [9]. In addition to cell migration [10, 11], SLK has been observed to play an important role in the breakdown of E-cadherin and ZO-1-positive junctions following TGFβ stimulation [12]. The loss of SLK delayed EMT, suggesting that it regulates the cytoskeletal changes associated with this process [12, 13]. We have previously shown that SLK is activated downstream of Neu (rat homolog of HER2) and requires PI3K or PLCγ activity for maximal activation [14]. Supporting a role for SLK in HER2-driven signaling, expression of a dominant negative SLK K63R reduced HER2-dependent chemotaxis in human breast cancer cell lines.

The Akt/PKB literature is constantly growing since the cloning of v-Akt in 1987 (extensively reviewed in [15]). It is now well established that Akt activation occurs downstream of phosphoinositide-3-kinase (PI3K), a lipid kinase implicated in tumorigenesis and the insulin response. The PI3K-dependent generation of PtdIns-3,4-P3 (PIP3) recruits and activates Akt at the membrane in concert with PDK1 and mTORC2. There are well over 100 reported Akt substrates implicated cell survival, proliferation, metabolism, neuronal functions, and angiogenesis. To date, the only transcription factors reported to be Akt targets are the Forkhead Box O proteins (FoxO1, 3, 4, and 6), regulating multiple transcription programs. Their phosphorylation induces their cytosolic retention through interactions with 14-3-3 proteins, blocking their access to target genes. The importance of turning off the PI3K-Akt pathway is underscored by the sheer number of negative feedback and cross-talk pathways. The most critical signal terminator is the phosphatase tumor suppressor PTEN, capable of converting the activator PIP3 to PIP2. Obviously, perturbations in AKT signaling lead to numerous pathological conditions such as overgrowth syndromes, autoimmune diseases, and cancer.

The Sry-HMG-box (Sox) family of transcription (reviewed in [16–18]) plays critical roles in many developmental processes. The SoxE group proteins, including Sox8, 9, and 10, have been extensively studied in the context of reproductive system development, neural crest cell-derived tissues, and cell types such as melanocytes. Although Sox9 and Sox10 have been widely studied during development, little is known about the mechanisms that regulate their activities other than their nucleocytoplasmic shuttling. The role of the Sox proteins in cancer progression remains elusive and controversial (reviewed in [18]). Different members have been shown to play a role both as tumor promoters and tumor suppressors in various types of cancers through the regulation of oncogenic pathways. Supporting this, Sox2 and Sox9 have been shown to be critical for the persistence of quiescent stem-like cancer cells through immune evasion [19]. We and others have recently identified Sox10 as a marker of triple-negative breast cancers (TNBC) [20] and secretory carcinomas, often triple negative and basal-like. Recently, Sox10 was found to be specifically expressed in mammary progenitor cells, including fetal and adult mammary cells in vivo [21] and be critical to maintain the stem cell state and reprogramming in breast cancer [22]. Strikingly, Sox10 deletion impairs mammary gland reconstitution whereas its overexpression increases it [21].

We have recently demonstrated that conditional SLK deletion in a MMTV-Neu background activates the PDK1-Akt system and accelerates breast tumor onset [23]. Although they are HER2/Neu+, those tumors display a basal-like phenotype. Strikingly, early lesions and tumor-derived cell lines express high levels of Sox10, a marker of TNBC [20]. This is accompanied by increased tumor stem cell activity in vitro and enhanced tumor growth in xenograft models. Interestingly, this phenotype is dependent on AKT activity and Sox10 expression. To gain insights into the molecular mechanisms regulating Sox10 expression, we have further assessed the role of the PI3K-PDK1-AKT pathway on Sox10 regulation. Our data show that AKT-driven Sox10 expression is dependent on Sox9 activation. Furthermore, this activation requires direct Sox9 phosphorylation by AKT. Our studies have uncovered Sox9 as a novel substrate for AKT, providing a novel link between AKT and tumor progression.

## Methods

### Plasmids and cloning

Fragments of the Sox10 promoter were amplified by PCR (see Supp. Table 1) from the bacterial artificial chromosome (BAC) RP23-424P8 which contains up to 12 kb of the Sox10 promoter and was purchased from the Centre of Applied Genomics (SickKids, Toronto, Canada). Each fragment of the Sox10 promoter was amplified using the indicated primers in Supplementary Table 1, digested with KpnI and XhoI (which were included in the forward or reverse primer, respectively) and ligated into pGL3P which was linearized with KpnI and XhoI.

pGEX-Sox9 was generated by PCR amplifying Sox9 cDNA from pCMV6-myc-DDK-Sox9 with the indicated primers in Supplementary Table 1, digested with BamHI and XhoI (which were included in the forward or reverse primer, respectively) and ligated into pGEX-4T2 which was linearized with BamHI and XhoI.

pGEX-Sox9^1-223^ was generated by linearizing and purifying pGEX-Sox9 with SmaI and NotI. The linear fragment was then blunted with Klenow (New England Biolabs) and purified from a 0.8% agarose gel using the QIAGEN Gel Extraction Kit (28704). The linearized vector was then incubated with T4 DNA ligase (New England Biolabs) to generate the circular plasmid.

Sox9 S181A mutants were generated using either pGEX-Sox9^1-223^ or pCMV6-myc-DDK-Sox9 as template. The point mutation was generated using the QuickChange XL Site-directed Mutagenesis Kit according to the manufacturer’s protocol. The primers used to generate the point mutations are listed in Supplementary Table 1.

### Cell culture

All mammary tumor cell lines were maintained in DMEM/F12 containing 10% FBS, 1% mammary epithelial growth supplement (Life Technologies), 1% penicillin/streptomycin, and 1% l-glutamine. Mammary tumor cell lines were isolated as described from individual tumors at endpoint [23]. All experiments were performed on three independent isolates and representative data are shown. All mammary tumor cell lines were used within 30 passages of initial isolation. All other cell lines used were maintained in DMEM containing 10% FBS, 1% penicillin/streptomycin, and 1% l-glutamine. Cells were cultured at 37°C in a humidified incubator set at 5% CO_2_. Cells were routinely passaged at a one in ten dilution every 2–3 days.

For plasmid and siRNA (Supplementary Table 3) transfections, cells were seeded such that they would reach approximately 75% confluency the day of the transfection. Lipofectamine 3000 was used for all transfections according to the manufacturer’s protocol using 8 μg of plasmid DNA or 200 nM of each siRNAs. Transfections were performed for 48–72 h prior to cell harvest.

### Immunohistochemistry

The tissues were harvested and fixed in 10% buffered formalin phosphate for 24 h and then transferred to 70% ethanol for storage. The tissues were formalin fixed, paraffin-embedded, and sectioned at a 5-μm thickness. Tissue sections were deparaffinized and subjected to antigen retrieval in 10 mM citrate buffer (pH 6.0) and quenched with 3% hydrogen peroxide. Sections were blocked in 5% donkey serum and incubated overnight with the indicated primary antibody (Supplementary Table 2) at 4°C. Sections were washed followed by incubation with the appropriate HRP-conjugated secondary antibody. For mouse monoclonal antibodies on mouse tissues, mouse-on-mouse blocking solution (Vector Laboratories) was added to the blocking step. All IHC staining were carefully controlled with no primary antibody controls. Staining was developed using DAB substrate (Sigma Aldrich), and sections were counterstained with hematoxylin. Sections were dehydrated in ethanol then xylene and mounted. Sections were imaged using the Aperio Scanscope (Leica Biosystems), and images were processed and/or analyzed using Imagescope or ImageJ. For quantification, stained and scanned sections were opened in ImageScope and the percentage of DAB-positive (brown) pixels for each core was quantified using the built-in Aperio positive pixel count algorithm. Only pixels that fell into the “strong positive” default setting were counted. For the murine hyperplastic lesions, a total of 10 mammary glands were surveyed for each genotype.

### Western blotting

Tissues and cell lines were homogenized in RIPA lysis buffer containing protease inhibitors (0.05% SDS, 1% Triton X-100, 1% NP-40, 50 mM Tris-HCl, pH 7.5, 150 mM NaCl, 2 mM EDTA, pH 8.0, 12 mM Na-Deoxycholate, 10 mM NaF, 1 mM DTT, 10 mM β-glycerophosphate, 0.6 mM NaVO_3_, 1 mM PMSF, 10 μg/mL leupeptin, 10 μg/mL aprotinin, 10 μg/mL pepstatin, and 100 μM benzamide). Lysate was cleared by centrifugation and protein concentrations were determined using Bradford reagent (Bio-Rad). Equal amounts of protein lysate were denatured and subject to SDS-PAGE. Samples were then transferred to a PVDF membrane and probed with the indicated primary antibody (Supplementary Table 2) overnight at 4°C in 5% BSA. The membranes were washed and incubated with the appropriate HRP-conjugated secondary antibody. Reactive proteins were detected by chemiluminescence (Perkin Elmer) and exposure to X-ray film.

### GST-fusion protein production and kinase assay

RP bacteria were transformed with the indicated GST or GST-fusion plasmids and glycerol stocks were stored at −80°C. Glycerol stocks were used to inoculate 3 mL of LB broth containing the appropriate antibiotic, and cultures were left to grow overnight at 37°C with shaking at 225 rpm. The 3-mL cultures were then expanded to 30 mL and left to shake at 225 rpm for 1 h at 37°C. GST-fusion protein production was then induced with 1 mM IPTG for 2 h at 37°C with shaking at 225 rpm. The bacterial cultures were then pelleted at 3000 rpm for 15 min at 4°C. Bacterial pellets were lysed in 500 μL of RIPA lysis buffer containing protease inhibitors and incubated on ice for 15 min with occasional vortexing. Lysates were then sonicated with three 15-s pulses and cleared by centrifugation at 14000 rpm for 30 min at 4°C. GST-fusion proteins were then purified with 20 μL of GST-sepharose beads (GE Healthcare) using the immunoprecipitation protocol.

In vitro kinase assays were performed as previously described [11]. Briefly, GST samples were pulled down and washed beads were resuspended in 1x kinase buffer (20-mM Tris-HCl, pH 7.4, 0.25-mM NaVO_3_, 1-mM NaF, 10-mM β-glycerophosphate, 1-mM DTT, and 15-mM MgCl_2_). The kinase assay was initiated by the addition of 1 μL of [^32^P]γATP (5 μCi/μL, Perkin Elmer) and incubated for 30 min at 30°C. Recombinant human GST-AKT fusion protein (200ng; Sigma Aldrich, SRP-5001) was added to the kinase assay master mix. The reactions were terminated with the addition of 4x SDS sample buffer (200-mM Tris-HCl, 400-mM DTT, 8% SDS, 0.4% bromophenol blue, and 40% glycerol) and subject to SDS-PAGE. The incorporation of the radiolabeled phosphate was detected by autoradiography and the efficiency of immunoprecipitation was determined by Western blot.

### Quantitative real-time PCR and microarray analysis

The total RNA was isolated using Trizol (Invitrogen). cDNA synthesis was performed using the Superscript III Reverse Transcriptase (Life Technologies) according to the manufacturer’s protocol. qRT-PCR was performed using an Applied Biosystems 7500 Real-Time Fast PCR thermocycler. Relative mRNA expression was calculated using the ΔΔC_T_ method and normalizing to total levels of ribosomal 18S. For microarray analysis, total RNA was hybridized to the Mouse Gene 2.0 ST Array (Affymetrix). Microarray data analysis was carried out in the *r* statistical programming environment with Bioconductor (GSE128514). Primers used for qRT-PCR are listed in Supplementary Table 1.

### Bisulfite sequencing

Genomic DNA for bisulfite conversion was isolated using the DNeasy Blood and Tissue Kit following the manufacturer’s protocol (QIAGEN). Bisulfite conversion of genomic DNA was performed using the EpiTect Plus DNA Bisulfite Kit (QIAGEN) following the manufacturer’s protocol. Promoter regions of control and bisulfite converted DNA were amplified using the primers indicated in Supplementary Table 1 using Taq polymerase (Invitrogen). Amplicons were subcloned into pGEM-T (Promega) according to the manufacturer’s protocol. Five clones for each conversion reaction were picked, miniprepped, and sequenced to identify methylated cytosine residues.

### Luciferase assay

For luciferase assays, 1.5 × 10^5^ cells were seeded in triplicate wells of a 6-well plate. The following day, the cells were transfected using lipofectamine 3000 with 1.25 μg of the appropriate pGL3P reporter construct and 1.25 μg of pRL-CMV (encoding Renilla luciferase) for 48 h. Luciferase assays were performed using the Dual Luciferase Assay Reporter System (Promega). Cells were collected in 1.5 mL Eppendorf tubes and lysed in 100 μL of 1X passive lysis buffer with rotation at room temperature for 30 min. Lysates were cleared by centrifugation at 13000 rpm for 10 min at room temperature. 20 μL of each sample was transferred in triplicate into a black-sided 96-well plate with a clear bottom. 100 μL of resuspended Luciferase Assay Substrate was added to each well and read using a luminometer with a read time of 10 s and a 2-s delay per well. 100 μL of Stop and Glo solution was then added to each well and read using a luminometer with a read time of 10 s and a 2-s delay per well. Luciferase counts were normalized to the pRL-CMV counts to account for differences in transfection efficiency between wells.

### Chromatin immunoprecipitation

For chromatin immunoprecipitation, 5×10^6^ cells from each condition were seeded in two 15-cm plates and cultured for 48 h. Cells were then cross-linked in 1% formaldehyde for 10 min at room temperature. The formaldehyde was then quenched with the addition of glycine to a final concentration of 125 mM. Nuclear extraction, chromatin digestion, and immunoprecipitation and DNA elution were all performed using the SimpleChip Kit (Cell Signaling Technologies) according to the manufacturer’s protocol with the addition of a pre-clearing step with beads alone prior to the immunoprecipitation. 10 μg of chromatin was used per immunoprecipitation with 1 μg of the indicated antibody. Following chromatin IP and DNA cleanup, 6.5 μL of chromatin was mixed with 35 μL of iTaq Universal SYBR Green Supermix (BioRad), 6.5 μL of the indicated primers (5μM), and 21 μL of nuclease-free water. The PCR reaction was started with an initial hold and denaturation at 50°C for 5 min and 95°C for 10 min, respectively, followed by 40 cycles of denaturation at 95°C for 15 s and Annealing and Extension at 60°C for 60 s. The percent input was then calculated as follows: percent input = 2% × 2^(C[T] 2% input sample – C[T] IP sample)^. The antibodies used for chromatin immunoprecipitation can be found in Supplementary Table 2. The primers used for ChIP-qPCR can be found in Supplementary Table 1.

## Results

### *Sox10* expression is regulated by several upstream promoter elements in SLK-deficient Neu-induced mammary tumor cells

We have previously shown that deletion of the Ste20-like kinase SLK induces Sox10 expression, enhancing tumorigenesis in vivo [23]. This is also accompanied by AKT upregulation and increased tumor stem/progenitor cell activity [23]. To gain further insights into the molecular mechanisms regulating Sox10 expression, we first assessed any potential epigenetic changes at the *SOX10* locus. The *SOX10* promoter has previously been shown to be epigenetically silenced in human gastric cancers and metastatic melanoma [24–26], the basal level of Sox10 expression is low in vitro, we hypothesized that *Sox10* gene induction could be due to gene demethylation in SLK^-/-^ NDL cells. We identified two putative CpG islands immediately upstream of the *Sox10* transcription start site using the MethPrimer CpG island prediction software (Supp. Figure 1A) [26]. To assess whether the *Sox10* gene was methylated, we treated SLK^fl/fl^ and SLK^-/-^ NDL cells with 5-aza-2’-deoxycytidine for 5 days to effectively eliminate any methylation marks within the genome. Following treatment, only a modest increase in *Sox10* mRNA was observed in the SLK^-/-^ NDL cells with no significant differences observed in SLK expressing controls (Supp. Figure 1B). The lack of a robust *Sox10* induction following 5-aza-2’-deoxycytidine treatment suggests that these putative CpG islands are not heavily methylated in either cell line. To validate this, we isolated genomic DNA and performed bisulfite sequencing on both putative CpG islands and compared the methylation pattern in SLK^fl/fl^ and SLK^-/-^ NDL cells. Using this approach, we found that only approximately 20% of cytosines within these CpG islands are methylated in either cell line with no significant differences observed following SLK deletion (Suppl. Figure 1C). Together, these results indicate that demethylation of the *Sox10* promoter is not the main mechanism of *Sox10* induction following SLK deletion.

**Fig. 1:**
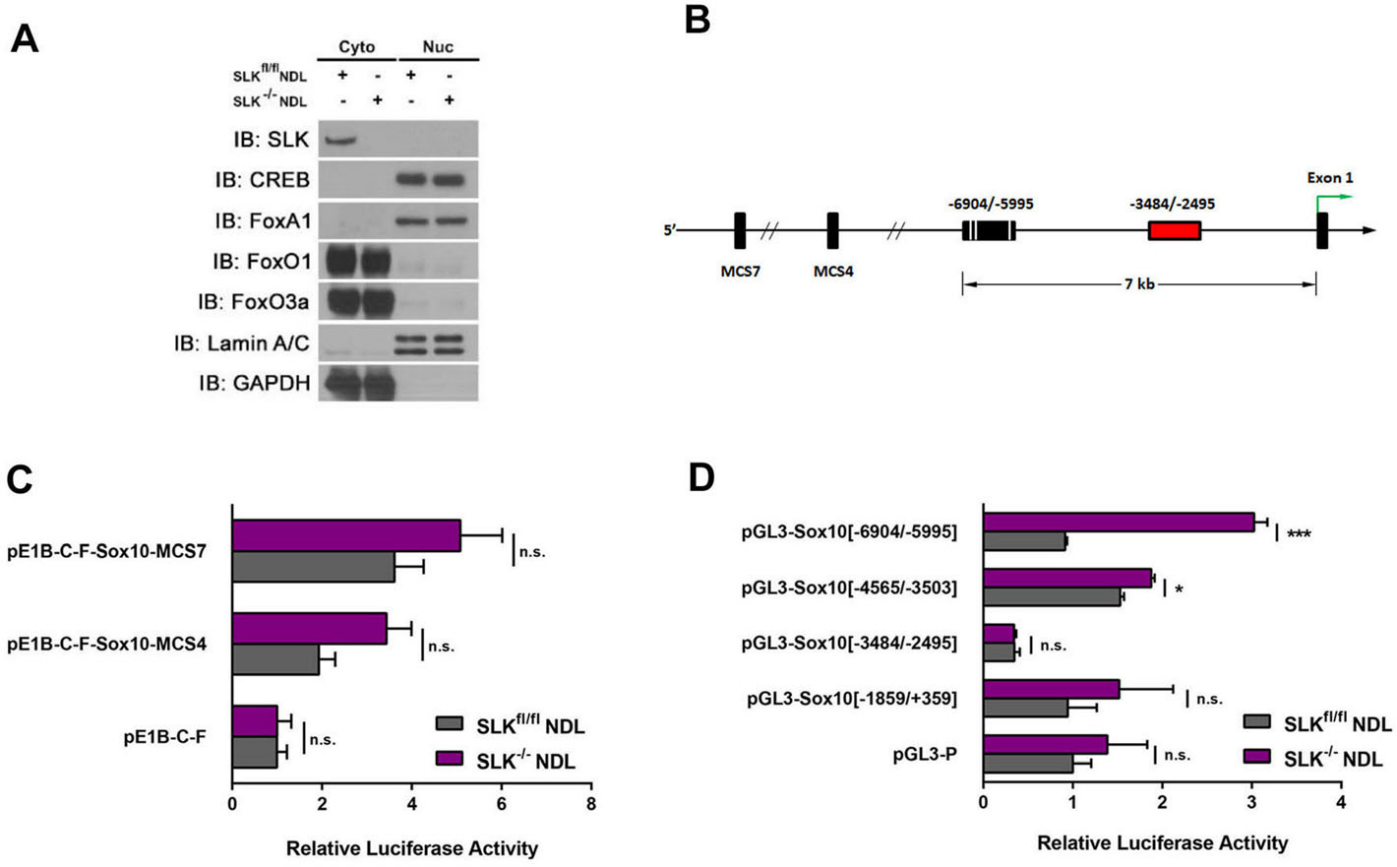
Identification of a differentially regulated fragment of the Sox10 promoter following *Slk* deletion. **a** SLK-null tumor cells do not differentially express known transcription factors that are AKT targets. Cytosolic (cyto) and nuclear (nuc) fractions were subjected to Western blot analysis for the indicated proteins. No changes were observed in levels or distribution of the AKT targets surveyed. **b** Schematic representation of putative SoxE binding sites within the Sox10 promoter. Luciferase assays from two multiple-species conserved enhancer sequences (MCS4 and 7) that have been shown to control *Sox10* expression (**c**) and approximately 1 kb fragments of the Sox10 promoter (**d**) assessed for their ability to drive luciferase activity in SLK^fl/fl^ and SLK^-/-^ NDL cells. Raw light units from the luciferase constructs were normalized to Renilla for each technical sample. Data is represented as the mean luciferase activity from three independent biological replicates +/− SEM. ns: no statistical difference, *p < 0.05, ***p < 0.0005

As epigenetic silencing is not responsible for the differential expression of *Sox10* in SLK^fl/fl^ and SLK^-/-^ NDL cells, we sought to identify potential signaling systems regulating *Sox10* expression. As we have shown that AKT activity is required for maintenance of *Sox10* expression in SLK knockout mammary tumor cells [23], we first assessed the levels and nuclear localization of known transcription factors that are AKT-responsive. Direct AKT targets implicated in transcription control include CREB and Forkhead box factors (FoxA1 and FoxO family [15]). Cellular fractionation and Western blot analysis (Fig. 1a) showed no difference in the levels and localization of those factors, suggesting that Sox10 is likely activated through other AKT-dependent mechanisms. To bootstrap our way back to transcriptional regulators controlling Sox10 expression in SLK-null cells, we analyzed potential regulatory regions around the *Sox10* gene. Comparative sequence analyses have previously identified multiple-species conserved sequences (MCS) that control *Sox10* expression [27]. As two of these MCS (4 and 7) have been shown to regulate *Sox10* expression in mammary epithelial cells (Fig. 1b for schematic), we tested whether these enhancer elements were differentially regulated in SLK^fl/fl^ and SLK^-/-^ NDL cells. Both enhancer elements drove luciferase activity above the levels of the control vector; however, no significant differences between cell lines was observed (Fig. 1c), suggesting that these enhancer sequences do not mediate the differential *Sox10* expression observed in the SLK^-/-^ NDL cells but perhaps mediate basal expression of *Sox10.*

Since little is known about the mechanisms regulating *Sox10* transcription other than the MCS sequences, we generated luciferase constructs driven by five independent Sox10 promoter elements within −7 kb of the transcription start site. Interestingly, the −3484/−2495 fragment of the promoter contains a repressive element which decreased luciferase activity by approximately 75% in both SLK^fl/fl^ and SLK^-/-^ NDL cell lines and may account for the lower basal expression of *Sox10* in cultured mammary epithelial tumor cell lines (Fig. 1d). Differential regulation of luciferase activity was observed in the two most distal promoter fragments between SLK^fl/fl^ and SLK^-/-^ NDL cells (Fig. 1d). The −6904/−5995 fragment showed a three-fold increase in luciferase activity in the SLK knockout cells, whereas that same element was inactive in the control cell line (Fig. 1d), suggesting that it harbors a differentially regulated enhancer region within the *Sox10* promoter.

### The Sox10 promoter contains putative SoxE binding sites which are bound by Sox9

**Fig. 2:**
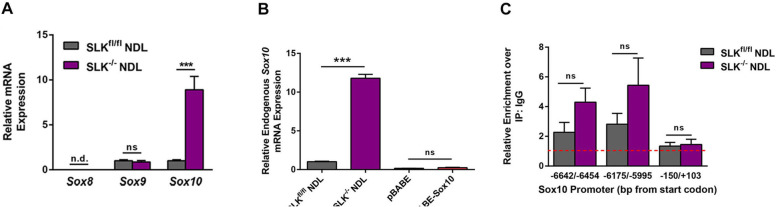
Sox9 binds directly to the SoxE binding sites within the Sox10 promoter. **a** The expression level of the SoxE family of transcription factors was assessed in SLK^fl/fl^ and SLK^-/-^ NDL cells by qRT-PCR analysis. **b** The levels of endogenous *Sox10* were assessed in the indicated cell lines using qRT-PCR primers located within the 3′UTR of Sox10. Note that the retroviral pBABE-Sox10 plasmid only contains the coding cDNA and not the 3′UTR. **c** Chromatin immunoprecipitation (ChIP) was performed on SLK^fl/fl^ and SLK^-/-^ NDL cells to assess Sox9 binding to the Sox10 promoter. Following Sox9 ChIP, qPCR analysis was performed across two putative SoxE binding sites within the −6904/−5995 fragment of the Sox10 promoter. qRT-PCR data was normalized to an IgG ChIP (dashed red line) or a negative control element within exon one (−150/+103). *N*=3, nd: no detectable expression, ns: no statistical difference, ***p < 0.0005

Interestingly, sequence analysis of the −6904/−5995 enhancer element revealed three consensus SoxE (Sox8, 9, and 10) binding sites [27] within this 909 base pair (bp) region (see Fig. 1c). However, Q-PCR analysis revealed no detectable expression of *Sox8* and no differences in *Sox9* expression in either cell lines grown in culture (Fig. 2a). As *Sox10* is highly expressed in the SLK-deficient cells, we investigated the possibility that Sox10 could control its own expression by assessing the levels of endogenous Sox10 in stable pBABE-Sox10 overexpressing SLK^fl/fl^ NDL cells. Using primers located in the 3′UTR, we found that exogenous Sox10 was unable to induce endogenous gene expression (Fig. 2b).

One possibility is that the locus is in an inactive topology or, alternatively, the Sox10 target sites are bound by another SoxE transcription factor. Interestingly, the Sox9 transcription factor has been shown to induce Sox10 gene expression through SoxE binding sites [16, 28–32], As *Sox8* is not expressed in NDL cells, we performed chromatin immunoprecipitation (ChIP) for Sox9 at the consensus SoxE binding sites within the active −6904/−5995 element in SLK-deficient NDL cells (Fig. 2c). Although we observed a significant enrichment for Sox9 above IgG pull down in both control and SLK knockout cells, no significant differences between the two cell lines were observed (Fig. 2c). Similar results were observed when using an anti-K27 acetylated histone H3 antibody, suggesting an open chromatin conformation around those enhancers (Suppl. Figure 2A). As a negative control, we also performed anti-Sox9 ChIP on a putative SoxE binding site within exon 1 that was unresponsive in our luciferase assay (Fig. 1d). This element was not bound by Sox9 in either cell line (Fig. 2c), suggesting that the enrichment observed within the −6904/−5995 element is not due to non-specific chromatin pull down by the Sox9 antibody. Importantly, these data show that the SoxE sites within the −6904/−5995 Sox10 genomic region are bound by Sox9, suggesting that it plays a role in Sox10 gene expression.

### *Slk* deletion increases Sox9 S181 phosphorylation

**Fig. 3:**
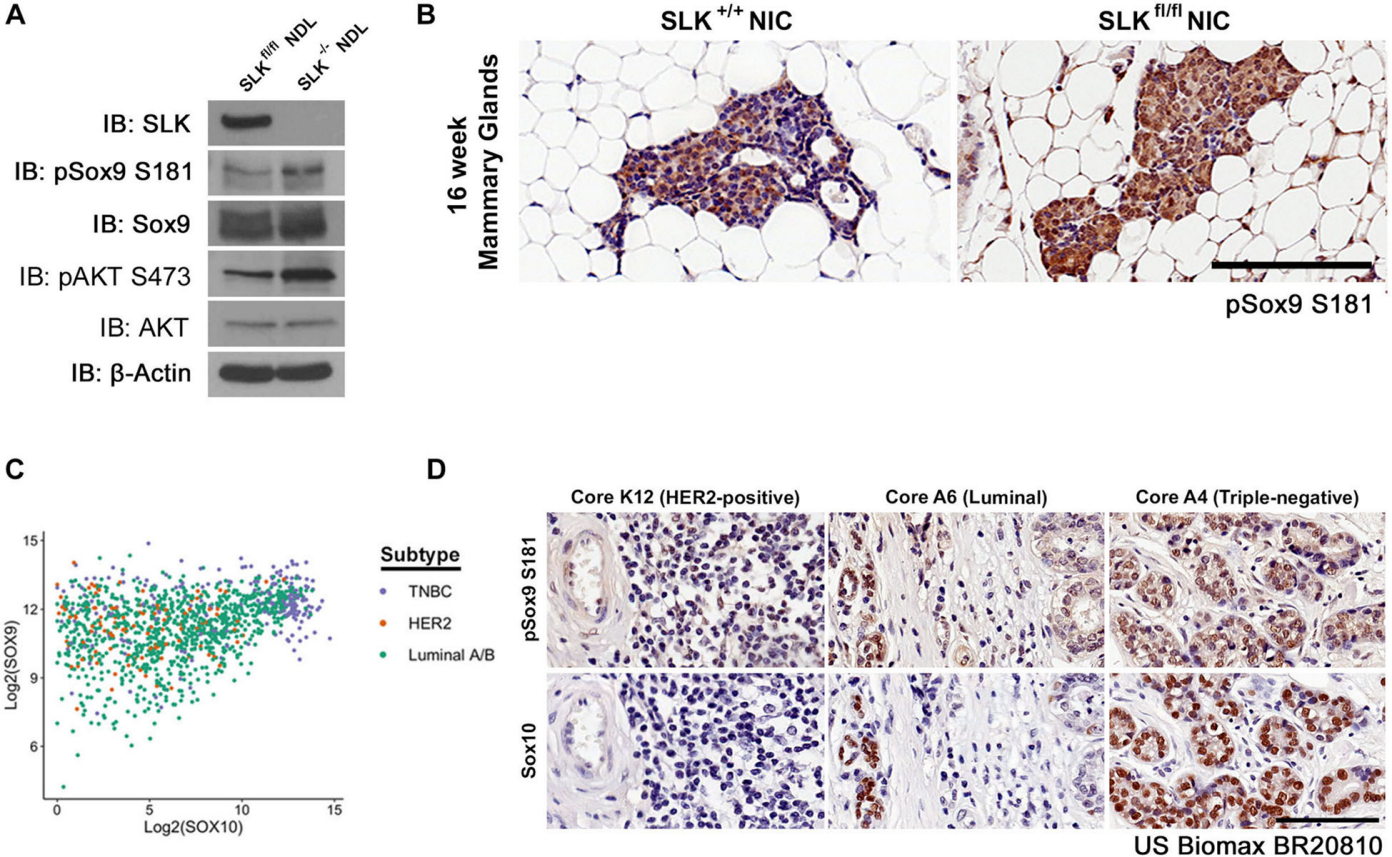
*Slk* deletion increases phosphorylated Sox9 which is correlated with higher Sox10 expression in human breast cancer samples. **a** Activity of Sox9 was assessed by Western blot analysis using a pS181-specific antibody in SLK^fl/fl^ and SLK^-/-^ NDL cells. Active AKT was assessed using a pS473 antibody. **b** pSox9 S181 histochemistry was performed on hyperplastic lesions from mammary glands of SLK^+/+^ and SLK^fl/fl^ NIC mice (*n*=10 glands/genotype) and representative images are shown for each genotype. Quantitation showed that SLK-null hyperplasia have a two-fold increase in pSox9 S181-positive nuclei (18 ± 4% vs 35 ± 7%; *p*<0.05). Scale bar = 100 μm. **c** The TCGA dataset was interrogated for expression of *SOX9* and *SOX10* across all breast cancer subtypes. Co-expression of both genes was observed in these patient samples with a Pearson correlation coefficient of 0.351 for all subtypes combined. **d** Serial sections of a human breast cancer TMA, BR20810, containing 104 breast cancer cases were stained with pSox9 S181 and Sox10 antibodies. Representative cores from each major molecular subtype, as defined by staining intensity of HER2, ESR1, and PGR, are shown. Scale bar = 200 μm

As Sox9 is bound to the Sox10 promoter in both wildtype and SLK knockout cell lines, we reasoned that the activity of Sox9 may be enhanced in the absence of SLK, through post-translational modifications, without affecting its DNA binding capacity. Interestingly, Sox9 has been shown to be phosphorylated at S64, S181, and S211 to regulate its nuclear import, stability, and DNA-binding/transcriptional activity [33–36]. Using phospho-serine 181 (pSox9 S181) as an indicator of Sox9 activity, we assessed the levels of pSox9 S181 in both cell lines. Western blot analysis showed a marked increase in the levels of Sox9 S181 phosphorylation in SLK knockout tumor cell lines that was correlated with elevated Sox10 levels in these cells (Fig. 3a), suggesting increased Sox9 activity in SLK-null tumor cells compared to the control. This was also correlated with higher levels of active AKT (pAKT S473; Fig. 3a). We have previously reported a two-fold increase in the number of Sox10+ nuclei from SLK-null mammary hyperplasia at 16 weeks of age [23]. Supporting this, immunohistochemical analysis for pSox9 S181 on hyperplastic lesions from SLK expressing and knockout hyperplastic lesions shows a similar increase (18 ± 4% vs 35 ± 7%) in the number of pSox9 S181-positive nuclei following SLK deletion in vivo (Fig. 3b). We did not observe any differences in the proportion of Sox10+ or pSox9 S181+ nuclei at endpoint, suggesting that SLK deletion affects tumor initiation as previously described [23]. Our previous analyses showed that SLK deletion results in a basal-like phenotype in MMTV-Neu mice [23]. Furthermore, as high as 13% of HER2+ patients fall within the *SLK-*low/*SOX10*-high subtype [23] and that Sox10 is a biomarker of the TNBC subtype [20]. Corroborating recent findings [37], analysis of those TCGA datasets reveals a strong correlation between high levels of Sox9 and Sox10 in TNBC samples (Fig. 3c, blue dots). To establish a potential link with active Sox9 in Sox10+ human samples, we assessed the levels of pSox9 S181 and Sox10 by immunohistochemistry across HER2-positive, luminal, and TNBC tumor cores. Consistent with our TCGA analysis (also see [23, 37]) and murine data, we observed that 43% of TNBC cores were Sox10^hi^ and that all Sox10^hi^ nuclei in those cores also showed a pSox9 S181^hi^ signal (Fig. 3d), suggesting that the activation of Sox9 and Sox10 induction is also observed in human breast cancer samples. Together, these studies suggest that the loss of SLK results in the activation of Sox9 that can directly induce Sox10 expression in murine tumors as well as human breast cancers.

### Sox9 is directly phosphorylated by AKT at serine 181

**Fig. 4:**
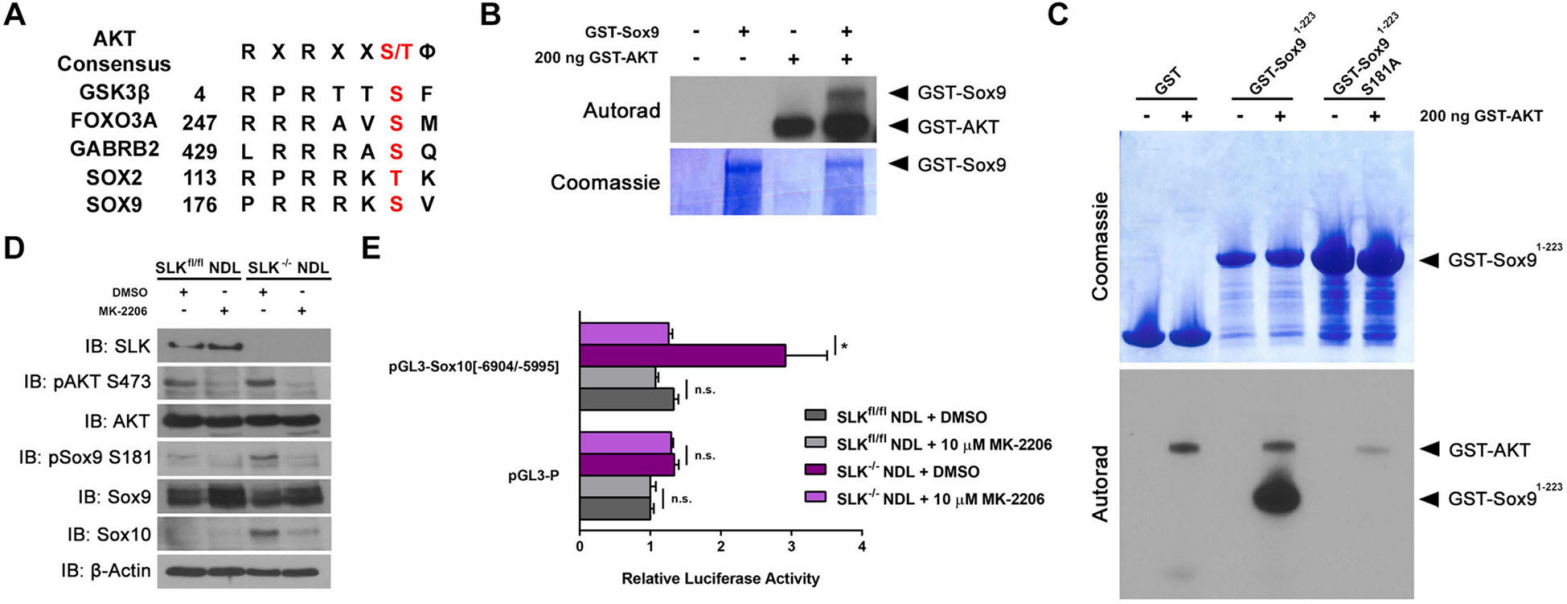
AKT directly phosphorylates Sox9 at serine 181. **a** The consensus AKT target sequence is provided aligned to bona fide AKT substrates (GSK-3b, FOXO3A, GABRB2, and SOX2) and the putative consensus sequence within SOX9 at serine 181. Within the consensus sequence X represents any amino acid, the phosphorylated serine (S) or threonine (T) is in red and ϕ is any hydrophobic residue. A recombinant kinase assay was performed using 200 ng of recombinant active GST-AKT and GST-Sox9 as substrate (**b**) or a GST-Sox9 S181A mutant (**c**). **d** Phosphorylation of Sox9 at serine 181 was assessed by Western blot analysis in SLK^fl/fl^ and SLK^-/-^ NDL cells treated with DMSO or MK-2206 for 72 hours. **e** Luciferase assays were performed in the presence or absence of MK-2206 using the responsive −6904/−5995 element of the Sox10 promoter. Raw light units from the luciferase constructs were normalized to Renilla for each technical sample. Data is represented as the mean luciferase activity from three independent biological replicates +/− SEM. ns: no statistical difference, *p < 0.05

We have previously shown that *Slk* deletion results in enhanced mammary tumorigenesis with the activation of PDK1 and AKT [23]. Therefore, we tested the possibility that Sox9 could be activated downstream of that pathway. Interestingly, amino acid sequence alignment and analysis of Sox9 revealed a putative AKT consensus phosphorylation site at serine 181, resembling several bona fide AKT targets (Fig. 4a). To assess whether Sox9 was a direct target of AKT, we performed in vitro kinase assays using recombinant proteins and monitored direct phosphorylation. As the full-length GST-Sox9 fusion protein is unstable and readily broken down in bacteria (not shown), we tested whether Sox9 was directly phosphorylated at serine 181 by AKT using a GST-Sox9^1-223^ truncation. Recombinant AKT was able to efficiently phosphorylate the GST-Sox9^1-223^ fusion protein, which contained the serine 181 residue (Fig. 4b, c). Mutation of serine 181 to alanine (S181A) in the GST-Sox9^1-223^ truncation completely abolished AKT-dependent phosphorylation of Sox9 (Fig. 4c), suggesting that Sox9 is a novel substrate for AKT at the serine 181 consensus site.

Supporting our in vitro kinase assays, treatment of SLK knockout cells with the AKT inhibitor MK-2206 significantly reduced the expression of Sox10 and the phosphorylation of AKT and Sox9 (Fig. 4d). Furthermore, MK-2206 treatment was sufficient to abolish luciferase activity driven from the −6904/−5995 enhancer element of the Sox10 promoter in SLK knockout cells (Fig. 4e). Knockdown of AKT1, 2 or 3 showed that a ~50% knockdown of AKT1 reduced Sox10 levels by about 50%. However, knockdown of AKT2 by 50–70% reduced Sox10 levels to about 20% of controls. Knockdown of AKT3 did not show a marked downregulation of Sox10. Together, these data suggest that AKT2 preferentially regulates Sox10 expression and that direct AKT-mediated activation of Sox9 regulates Sox10 transcription from the −6904/−5995 enhancer element.

### Phosphorylation of Sox9 at serine 181 is required for maximal transcriptional activity

**Fig. 5:**
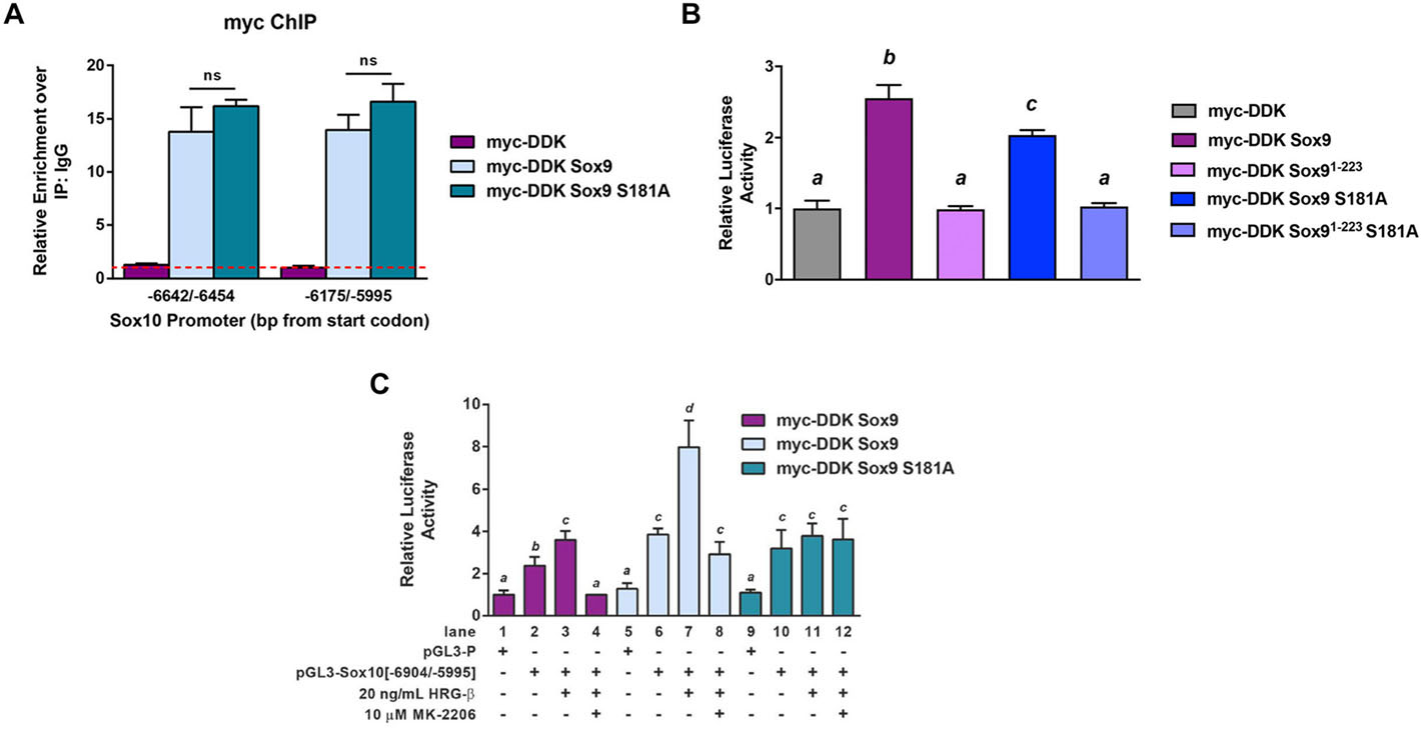
Sox9 phosphorylation at serine 181 is required for maximal transcriptional activation of the Sox10 promoter. **a** Anti-myc ChIP was performed on SLK^-/-^ NDL cells transiently transfected with myc-DDK, myc-DDK-Sox9, or myc-DDK-Sox9 S181A. Following anti-myc ChIP, qPCR analysis was performed across two SoxE binding sites within the −6904/−5995 fragment of the Sox10 promoter. qRT-PCR data was normalized to an IgG ChIP (dashed red line). ns: no statistical difference. **b** Luciferase assays were performed on SLK^-/-^ NDL cells that were transiently transfected with the indicated plasmids along with the responsive −6904/−5995 element of the Sox10 promoter. Raw light units from the luciferase constructs were normalized to Renilla for each technical sample. Data is represented as the mean luciferase activity from three independent biological replicates +/− SEM. All bars with the same letter are not statistically significant from each other. *a* compared to *b*: *p* < 0.005; *a* compared to *c*: *p* < 0.005; *b* compared to *c*: *p* < 0.05. **c** SLK^-/-^ NDL cell were transfected with the indicated plasmids along with the responsive −6904/−5995 element of the Sox10 promoter for 48 h. Cells were treated with heregulin-β (HRG-β), AKT inhibitor (MK-2206), or vehicle control for 2 h prior collecting the cell lysate. Raw light units from the luciferase constructs were normalized to Renilla for each technical sample. Data is represented as the mean luciferase activity from three independent biological replicates +/− SEM. All bars with the same letter are not statistically significant from each other. *a* compared to *b*: *p*<0.05, *a* compared to *c* or *d*: *p*<0.005, *b* compared to *c*: *p*<0.05, *b* compared to *d*: *p*<0.005 and *c* compared to *d*: *p*<0.05

To assess the effect of Sox9 phosphorylation on Sox10 transcriptional regulation, we first tested whether phosphorylation of Sox9 was required for direct binding to the Sox10 promoter. SLK^-/-^ NDL cells were transfected with an empty vector, myc-Sox9 or myc-Sox9 S181A and subjected to anti-myc ChIP on the Sox10 promoter (Fig. 5a). No differences were observed in DNA binding activity for the Sox9 S181A mutant (Fig. 5a), suggesting that S181 phosphorylation is not required for enhancer binding. Next, we investigated whether Sox9 S181A expression could impair luciferase expression driven from the Sox10 promoter. Transfection of myc-Sox9 into SLK^-/-^ NDL cells with the Sox10 −6904/−5995 enhancer element resulted in a 2.5-fold increase in luciferase activity above a Myc control vector and myc-Sox9^1-223^, lacking the C-terminal transactivation domain (Fig. 5b). Albeit significantly lower than wildtype Sox9, overexpression of the Sox9 S181A mutant also resulted in an increase in luciferase activity (Fig. 5b). As Sox9 can homodimerize when bound to DNA [38], one possibility is that the Sox9 S181A mutant can form a complex with endogenous Sox9, resulting in reduced transcriptional activity imparted by the mutant Sox9.

Since Sox9 is present on the Sox10 promoter of both cell lines, we hypothesized that the activation of AKT results in DNA-bound Sox9 phosphorylation and increased transcriptional activity. To test this, we transiently overexpressed myc, myc-Sox9, or myc-Sox9 S181A in SLK^-/-^ NDL cells and performed luciferase assays using the −6904/−5995 fragment of the Sox10 promoter. Consistent with our previous data, we observed a two- to three-fold increase in luciferase activity from the Sox10 promoter fragment with the Myc vector control (Fig. 5c) lanes 1 and 2). Additionally, we observed an AKT-dependent increase in luciferase activity following heregulin-stimulation in these cells (Fig. 5c, lanes 3 and 4). These controls further support the notion that *Sox10* induction from the −6904/−5995 promoter element is dependent on Neu signaling through AKT. To validate that these signals are dependent on Sox9 transcriptional activity, we assessed luciferase activity in the presence of myc-Sox9 or a myc-Sox9 S181A mutant with reduced transcriptional activity [34]. As observed in Fig. 5b, in unstimulated cells, both wildtype and mutant Sox9 were sufficient to increase luciferase activity four-fold above background (Fig. 5c, lanes 5 and 6 compared to lanes 9 and 10). However, following stimulation with heregulin, a two-fold increase in luciferase activity was observed in myc-Sox9 expressing cells, whereas no significant change was observed in cells expressing the myc-Sox9 S181A mutant (Fig. 5c, lanes 6 and 7 compared to lanes 10 and 11). As for myc control-transfected cells, the increased luciferase activity following heregulin-stimulation in myc-Sox9 transfected cells was completely dependent on AKT activity (Fig. 5c, lanes 7 and 8). Together, these data show that the −6904/−5995 region of the Sox10 promoter is an AKT-responsive element which requires the transcriptional activity of Sox9. Furthermore, our results suggest that Sox9 S181 phosphorylation does not affect DNA binding but is required for maximal transcriptional activity on this Sox10 enhancer.

## Discussion

Here, we have shown that the observed induction of Sox10 in SLK^-/-^ NDL tumor cells is dependent on a novel enhancer located at about −7kb upstream of the putative Sox10 start site. Our data show that Sox10 expression is also directly correlated with Sox9 phosphorylation and activity in our SLK^-/-^ NDL mouse model and human tissue microarrays. We demonstrate that AKT can directly phosphorylate DNA-bound Sox9 at serine 181, increasing its transcriptional activity on the −7kb Sox10 enhancer. These data also extends the growing list of AKT substrates.

Our data demonstrate that an AKT-dependent pathway regulates *Sox10* transcription specifically through an enhancer fragment located between −6904 and −5995 from the putative start site. Scanning of this enhancer revealed three SoxE sites. Although we have observed some variability, no significant differences were found in Sox9 binding to those SoxE sites, suggesting that Sox9 is bound equally to these elements in both SLKfl/fl and SLK^-/-^ NDL cells. This suggests that Sox9 phosphorylation at S181 activates transcription without altering its DNA binding activity. Given the large increase in Sox10 expression in the SLK^-/-^ cells, it is likely that, in addition to Sox9 binding to SoxE sites, other transcriptional mechanisms are activated. One possibility is that phosphorylation at this site is required to recruit transcriptional cofactors which may be required for maximal activity.

Using amino acid alignment and kinase assays, we have shown that AKT can directly phosphorylate Sox9 in vitro and that enhancer activity is lost following AKT inhibition (Fig. 4). This suggests that the inability for AKT to phosphorylate Sox9 prevents its activation and Sox10 induction. Although we predict that AKT directly phosphorylates Sox9 at serine 181, this remains to be demonstrated in vivo. However, this may be more challenging as a number of other kinases have been shown to phosphorylate that site in other contexts [33–36]. Alternatively, it is possible that AKT directly phosphorylates and inhibits the activity of an unknown phosphatase that targets pSox9 S181. It is also possible that this phosphorylation may be mediated by another kinase in the AKT pathway such as S6 kinase (S6K). S6 Kinase is activated by mTOR downstream of AKT and has a consensus phosphorylation motif matching that of Sox9 S181 [39]. Combined with in vitro kinase assays, rapamycin treatment to inhibit mTOR would address the potential role of S6K in Sox9 activation and *Sox10* expression.

To validate the role of Sox9 in regulating the transcription of *Sox10*, we performed luciferase assays using the AKT-responsive element from the Sox10 promoter. Surprisingly, myc-Sox9 or the myc-Sox9 S181A mutant is both capable of activating luciferase expression from the −7kb enhancer under basal conditions (Fig. 5). This was also observed for the collagen II promoter, where the induction by both Sox9 constructs is relatively high above background [34]. In the context of the collagen II promoter, wildtype Sox9 was sufficient to boost luciferase activity following co-transfection with the catalytic subunit of PKA, whereas the Sox9 S181A mutant failed to do so [34]. Similarly, treatment of the myc-Sox9 transfected cells with heregulin increased luciferase activity two-fold above the untreated sample (Fig. 5c, lanes 6 and 7). However, expression of the phospho-deficient mutant abrogated this effect (Fig. 5c, lanes 10 and 11). One possibility is that Sox9 activity through phosphorylation at S181 and/or S211 can be further increased by specific signals while bound to DNA as a homodimer. This would explain the slight reduction in enhancer activity when a myc-Sox9 S181A is expressed in SLK^-/-^NDL cells. The S181A mutant may have a dominant negative effect on a Sox9/Sox9 S181A dimer.

We have previously shown that Sox10 is biomarker for the TNBC subtype and is induced following the mammary gland-specific deletion of SLK in a MMTV-Neu mouse model, inducing a basal-like phenotype in a HER2+ model [23]. The induction of Sox10 is accompanied by increased stemness and accelerated tumorigenesis [23]. Recently, Sox10 has been shown to be expressed in mammary progenitor cells in vivo [21] and be critical to maintain stemness in breast cancer [22]. Interestingly, Sox2 and Sox9 have been shown to be critical for the persistence of quiescent stem-like breast cancer cells [19]. It is not clear whether those Sox9+ stem-like cells also express Sox10.

Supporting our findings in murine mammary tumors, we have also observed a correlation between pSox9 S181 levels and Sox10 expression in a proportion of HER2+ human tumor samples [23]. In addition, Fig. 3c shows that a high proportion of Sox10^hi^ TNBC samples also show a Sox9^hi^ phenotype (blue dots in the SOX9/SOX10 high quadrant of the data set), suggesting that high Sox9 activity could occur without increased Akt activity. Similarly, Sox9 is highly phosphorylated in human triple-negative breast cancers expressing high levels of Sox10 (see Fig. 3 and [37]). Therefore, it is possible that a similar mechanism exists in breast cancers that display high level of PI3K/AKT pathway activation. In fact, oncogenic activation of the PI3K/AKT signaling pathway is frequent in TNBC and most commonly occurs following *PIK3CA* gain-of-function mutations or *P53* inactivation [40, 41]. Treatment of triple-negative breast cancer cell lines with the allosteric AKT inhibitor, MK-2206, inhibits tumor growth and increases sensitivity to other chemotherapeutic agents [42–44]. Clinical trials for TNBC have shown that AKT inhibitors, including MK-2206, have a synergistic effect with paclitaxel and significantly improve progression-free and overall survival [42, 43]. In addition to regulating cell survival and promoting tumor growth, we have shown that AKT controls the expression of *Sox10*. Therefore, we believe that the therapeutic targeting of AKT could decrease mammary tumor stem/progenitor activity by downregulating *SOX10* expression in TNBC.

A recent murine model for TNBC has identified a high frequency of both *Egfr* and *Fgfr2* amplifications [21, 30]. FGF-signaling has previously been shown to induce the expression of both *Sox9* and *Sox10* [21, 30]. In light of our observations, *Fgfr2* amplifications in TNBC [41] may be sufficient to upregulate *Sox9* expression. Combined with gain-of-function mutations in *PIK3CA*, this would be sufficient to increase Sox9 activity in an AKT-dependent manner. Therefore, the combinatorial treatment of triple-negative breast cancers with FGFR and AKT inhibitors may target two distinct signaling pathways that drive *SOX10* expression by blocking both the induction and activation of Sox9.

One of the largest barriers to the effective treatment of HER2-positive breast cancers is the rapid acquisition of Herceptin-resistance [45, 46] often accompanied by significantly elevated levels of phosphorylated active AKT [47]. As both chronic Herceptin treatment and SLK deletion result in the hyperactivation of AKT, we speculate that these Herceptin-resistant tumors may have also acquired *SOX10* expression. Therefore, it is possible that Herceptin-resistant tumors with high levels of AKT activity may be dependent on the oncogenic and stem/progenitor activities of Sox10.

## Conclusions

We and others have reported that triple-negative and basal/stem-like breast cancers can be defined by a high level of *SOX10* expression [20, 48–51]. Here, we have uncovered a novel link between constitutive activation of AKT, Sox9 phosphorylation, and the induction of *Sox10* gene expression. Our studies have identified Sox9 as a novel AKT substrate. As recently highlighted [20, 22], Sox10 transcriptional reprogramming may be a hallmark of TNBCs. This raises the possibility that targeted therapies to the Sox9-Sox10 axis could represent an important first step in the treatment of TNBCs. This might also have major implications in the targeting of tumor stem cells in TNBCs and our understanding of the role of Sox9 and Sox10 during development.

## Supplementary Information

**Additional file 1: Supplementary Figure 1.** Induction of *Sox10* following *Slk* deletion is not due to promoter demethylation. **A,** Schematic representation of the murine *Sox10* gene. The transcriptional start site is indicated by the forward arrow. The location of all CG dinucleotides are represented as individual vertical lines. Methprimer CpG prediction software was used to identify potential CpG islands and are shown as the underlined regions. **B,** SLK^fl/fl^ and SLK^-/-^ NDL cells were treated with 5-aza-2’-deoxycytidine (5’-Aza) for five days. The levels of *Sox10* transcript was assessed by qPCR analysis. **C,** Bisulfite sequencing of genomic DNA from SLK^fl/fl^ and SLK^-/-^ NDL cells was performed. Five independent clones from each cell line was sequenced. A representative plot of unmethylated (open) and methylated (filled) CpG repeats from two putative CpG islands identified in (**A**) is presented. ns: no statistical difference, * p < 0.05.

**Additional file 2: Supplementary Figure 2.** Sox10 induction is preferentially mediated by AKT2. (**A**) Chromatin immunoprecipitation (ChIP) was performed on SLK^fl/fl^ and SLK^-/-^ NDL cells to assess K27 Acetylated histone H3 binding to the Sox10 enhancers. Following ChIP, qPCR analysis was performed across two putative SoxE binding sites within the -6904/-5995 fragment of the Sox10 promoter. qRT-PCR data was normalized to an IgG ChIP or a negative control element within exon one (-150/+103) as in Fig. 2. No statistical differences were observed between the cell lines. *N*=3. (**B**) Knock down of AKT1 or AKT2 results in Sox10 downregulation. AKT1 knockdowns were ~50% at best with two independent siRNAs. AKT2 knockdowns with two siRNAs ranged from 50-70% with a marked downregulation (80-90%) of Sox10. (**C**) AKT3 knockdowns from 40-60% did not show any appreciable reduction in Sox10 levels. Underexposed blots were subjected to ImageJ densitometry and normalized to β-actin and NT controls. NT= non targeting siRNA control.

**Additional file 3: Table S1.** List of Primers.

**Additional file 4: Table S2.** Antibodies.

**Additional file 5: Table S3.** Akt siRNAs (Sigma).

## Data Availability

The datasets generated and/or analyzed during the current study are available in The Cancer Genome Atlas (v2, accessed in 2019) repository [https://www.cancer.gov/types/breast].

## Abbreviations

MMTV: Mouse mammary tumor virus
ChIP: Chromatin immunoprecipitation
EMT: Epithelial to mesenchymal transition
PIP: Phosphatidyl-inositol phosphate
TNBC: Triple negative breast cancer
NDL: Neu deleted line

## References

[1] Perou CM, Sorlie T, Eisen MB, van de Rijn M, Jeffrey SS, Rees CA, Pollack JR, Ross DT, Johnsen H, Akslen LA (2000). Molecular portraits of human breast tumours. Nature.

[2] Sorlie T, Perou CM, Tibshirani R, Aas T, Geisler S, Johnsen H, Hastie T, Eisen MB, van de Rijn M, Jeffrey SS, Thorsen T, Quist H, Matese JC, Brown PO, Botstein D, Lonning PE, Borresen-Dale AL (2001). Gene expression patterns of breast carcinomas distinguish tumor subclasses with clinical implications. Proc Natl Acad Sci U S A.

[3] Mansour EG, Ravdin PM, Dressler L (1994). Prognostic factors in early breast carcinoma. Cancer.

[4] Guy CT, Webster MA, Schaller M, Parsons TJ, Cardiff RD, Muller WJ (1992). Expression of the neu protooncogene in the mammary epithelium of transgenic mice induces metastatic disease. Proc Natl Acad Sci U S A.

[5] Dankort DL, Wang Z, Blackmore V, Moran MF, Muller WJ (1997). Distinct tyrosine autophosphorylation sites negatively and positively modulate neu-mediated transformation. Mol Cell Biol.

[6] Gullick WJ, Srinivasan R (1998). The type 1 growth factor receptor family: new ligands and receptors and their role in breast cancer. Breast Cancer Res Treat.

[7] Harari D, Yarden Y (2000). Molecular mechanisms underlying ErbB2/HER2 action in breast cancer. Oncogene.

[8] Menard S, Tagliabue E, Campiglio M, Pupa SM (2000). Role of HER2 gene overexpression in breast carcinoma. J Cell Physiol.

[9] Al-Zahrani KN, Baron KD, Sabourin LA (2013). Ste20-like kinase SLK, at the crossroads: a matter of life and death. Cell Adh Migr.

[10] Quizi JL, Baron K, Al-Zahrani KN, O'Reilly P, Sriram RK, Conway J, Laurin AA, Sabourin LA (2013). SLK-mediated phosphorylation of paxillin is required for focal adhesion turnover and cell migration. Oncogene.

[11] Wagner S, Storbeck CJ, Roovers K, Chaar ZY, Kolodziej P, McKay M, Sabourin LA (2008). FAK/src-family dependent activation of the Ste20-like kinase SLK is required for microtubule-dependent focal adhesion turnover and cell migration. PLoS One.

[12] Conway J, Al-Zahrani KN, Pryce BR, Sabourin LA (2017). Transforming growth factor ß-induced epithelial to mesenchymal transition requires the Ste20-like kinase SLK independently of its catalytic activity. Oncotarget.

[13] Wang K, Hong RL, Lu JB, Wang DL (2018). Ste20-like kinase is upregulated in glioma and induces glioma invasion. Neoplasma.

[14] Roovers K, Wagner S, Storbeck CJ, O'Reilly P, Lo V, Northey JJ, Chmielecki J, Muller WJ, Siegel PM, Sabourin LA (2009). The Ste20-like kinase SLK is required for ErbB2-driven breast cancer cell motility. Oncogene.

[15] Manning BD, Toker A (2017). AKT/PKB signaling: navigating the network. Cell.

[16] Bondurand N, Sham MH (2013). The role of SOX10 during enteric nervous system development. Dev Biol.

[17] She ZY, Yang WX (2015). SOX family transcription factors involved in diverse cellular events during development. Eur J Cell Biol.

[18] Xu YR, Yang WX (2017). SOX-mediated molecular crosstalk during the progression of tumorigenesis. Semin Cell Dev Biol.

[19] Malladi S, Macalinao DG, Jin X, He L, Basnet H, Zou Y, de Stanchina E, Massague J (2016). Metastatic latency and immune evasion through autocrine inhibition of WNT. Cell.

[20] Al-Zahrani KN, Cook DP, Vanderhyden BC, Sabourin LA (2018). Assessing the efficacy of androgen receptor and Sox10 as independent markers of the triple-negative breast cancer subtype by transcriptome profiling. Oncotarget.

[21] Dravis C, Spike BT, Harrell JC, Johns C, Trejo CL, Southard-Smith EM, Perou CM, Wahl GM (2015). Sox10 regulates stem/progenitor and mesenchymal cell states in mammary epithelial cells. Cell Rep.

[22] Dravis C, Chung CY, Lytle NK, Herrera-Valdez J, Luna G, Trejo CL, et al. Epigenetic and transcriptomic profiling of mammary gland development and tumor models disclose regulators of cell state plasticity. Cancer Cell. 2018;34(3):466–82. 10.1016/j.ccell.2018.08.001 PMC6152943 30174241

[23] Al-Zahrani KN, Abou-Hamad J, Cook DP, Pryce BR, Hodgins JJ, Labreche C, Robineau-Charette P, de Souza CT, Bell JC, Auer RC (2020). Loss of the Ste20-like kinase induces a basal/stem-like phenotype in HER2-positive breast cancers. Oncogene.

[24] Tong X, Li L, Li X, Heng L, Zhong L, Su X, Rong R, Hu S, Liu W, Jia B, Liu X, Kou G, Han J, Guo S, Hu Y, Li C, Tao Q, Guo Y (2014). SOX10, a novel HMG-box-containing tumor suppressor, inhibits growth and metastasis of digestive cancers by suppressing the Wnt/beta-catenin pathway. Oncotarget.

[25] Jin SG, Xiong W, Wu X, Yang L, Pfeifer GP (2015). The DNA methylation landscape of human melanoma. Genomics.

[26] Li LC, Dahiya R (2002). MethPrimer: designing primers for methylation PCRs. Bioinformatics.

[27] Antonellis A, Huynh JL, Lee-Lin SQ, Vinton RM, Renaud G, Loftus SK, Elliot G, Wolfsberg TG, Green ED, McCallion AS (2008). Identification of neural crest and glial enhancers at the mouse Sox10 locus through transgenesis in zebrafish. PLoS Genet.

[28] Betancur P, Bronner-Fraser M, Sauka-Spengler T (2010). Genomic code for Sox10 activation reveals a key regulatory enhancer for cranial neural crest. Proc Natl Acad Sci U S A.

[29] Betancur P, Sauka-Spengler T, Bronner M (2011). A Sox10 enhancer element common to the otic placode and neural crest is activated by tissue-specific paralogs. Development.

[30] Chen Z, Huang J, Liu Y, Dattilo LK, Huh SH, Ornitz D, Beebe DC (2014). FGF signaling activates a Sox9-Sox10 pathway for the formation and branching morphogenesis of mouse ocular glands. Development.

[31] Dutton JR, Antonellis A, Carney TJ, Rodrigues FS, Pavan WJ, Ward A, Kelsh RN (2008). An evolutionarily conserved intronic region controls the spatiotemporal expression of the transcription factor Sox10. BMC Dev Biol.

[32] Werner T, Hammer A, Wahlbuhl M, Bosl MR, Wegner M (2007). Multiple conserved regulatory elements with overlapping functions determine Sox10 expression in mouse embryogenesis. Nucleic Acids Res.

[33] Coricor G, Serra R (2016). TGF-beta regulates phosphorylation and stabilization of Sox9 protein in chondrocytes through p38 and Smad dependent mechanisms. Sci Rep.

[34] Huang W, Zhou X, Lefebvre V, de Crombrugghe B (2000). Phosphorylation of SOX9 by cyclic AMP-dependent protein kinase A enhances SOX9's ability to transactivate a Col2a1 chondrocyte-specific enhancer. Mol Cell Biol.

[35] Kumar D, Lassar AB (2009). The transcriptional activity of Sox9 in chondrocytes is regulated by RhoA signaling and actin polymerization. Mol Cell Biol.

[36] Malki S, Nef S, Notarnicola C, Thevenet L, Gasca S, Mejean C, Berta P, Poulat F, Boizet-Bonhoure B (2005). Prostaglandin D2 induces nuclear import of the sex-determining factor SOX9 via its cAMP-PKA phosphorylation. EMBO J.

[37] Ma Y, Shepherd J, Zhao D, Bollu LR, Tahaney WM, Hill J, Zhang Y, Mazumdar A, Brown PH (2020). SOX9 is essential for triple-negative breast cancer cell survival and metastasis. Mol Cancer Res.

[38] Huang YH, Jankowski A, Cheah KS, Prabhakar S, Jauch R (2015). SOXE transcription factors form selective dimers on non-compact DNA motifs through multifaceted interactions between dimerization and high-mobility group domains. Sci Rep.

[39] Pei JJ, An WL, Zhou XW, Nishimura T, Norberg J, Benedikz E, Gotz J, Winblad B (2006). P70 S6 kinase mediates tau phosphorylation and synthesis. FEBS Lett.

[40] Cossu-Rocca P, Orru S, Muroni MR, Sanges F, Sotgiu G, Ena S, Pira G, Murgia L, Manca A, Uras MG (2015). Analysis of PIK3CA mutations and activation pathways in triple negative breast cancer. PLoS One.

[41] Liu H, Murphy CJ, Karreth FA, Emdal KB, White FM, Elemento O, Toker A, Wulf GM, Cantley LC (2018). Identifying and targeting sporadic oncogenic genetic aberrations in mouse models of triple-negative breast cancer. Cancer Discov.

[42] Costa RLB, Han HS, Gradishar WJ (2018). Targeting the PI3K/AKT/mTOR pathway in triple-negative breast cancer: a review. Breast Cancer Res Treat.

[43] Kim SB, Dent R, Im SA, Espie M, Blau S, Tan AR, Isakoff SJ, Oliveira M, Saura C, Wongchenko MJ (2017). Ipatasertib plus paclitaxel versus placebo plus paclitaxel as first-line therapy for metastatic triple-negative breast cancer (LOTUS): a multicentre, randomised, double-blind, placebo-controlled, phase 2 trial. Lancet Oncol.

[44] Sangai T, Akcakanat A, Chen H, Tarco E, Wu Y, Do KA, Miller TW, Arteaga CL, Mills GB, Gonzalez-Angulo AM, Meric-Bernstam F (2012). Biomarkers of response to Akt inhibitor MK-2206 in breast cancer. Clin Cancer Res.

[45] Gajria D, Chandarlapaty S (2011). HER2-amplified breast cancer: mechanisms of trastuzumab resistance and novel targeted therapies. Expert Rev Anticancer Ther.

[46] Wolff AC, Hammond ME, Schwartz JN, Hagerty KL, Allred DC, Cote RJ, Dowsett M, Fitzgibbons PL, Hanna WM, Langer A, McShane L, Paik S, Pegram MD, Perez EA, Press MF, Rhodes A, Sturgeon C, Taube SE, Tubbs R, Vance GH, van de Vijver M, Wheeler TM, Hayes DF, American Society of Clinical Oncology/College of American Pathologists (2007). American Society of Clinical Oncology/College of American Pathologists guideline recommendations for human epidermal growth factor receptor 2 testing in breast cancer. Arch Pathol Lab Med.

[47] Chan CT, Metz MZ, Kane SE (2005). Differential sensitivities of trastuzumab (Herceptin)-resistant human breast cancer cells to phosphoinositide-3 kinase (PI-3K) and epidermal growth factor receptor (EGFR) kinase inhibitors. Breast Cancer Res Treat.

[48] Cimino-Mathews A, Subhawong AP, Elwood H, Warzecha HN, Sharma R, Park BH, Taube JM, Illei PB, Argani P (2013). Neural crest transcription factor Sox10 is preferentially expressed in triple-negative and metaplastic breast carcinomas. Hum Pathol.

[49] Feng W, Liu S, Zhu R, Li B, Zhu Z, Yang J, Song C (2017). SOX10 induced Nestin expression regulates cancer stem cell properties of TNBC cells. Biochem Biophys Res Commun.

[50] Krings G, Joseph NM, Bean GR, Solomon D, Onodera C, Talevich E, Yeh I, Grenert JP, Hosfield E, Crawford ED, Jordan RC, van Zante A, Zaloudek C, Shin SJ, Chen YY (2017). Genomic profiling of breast secretory carcinomas reveals distinct genetics from other breast cancers and similarity to mammary analog secretory carcinomas. Mod Pathol.

[51] Min L, Zhang C, Qu L, Huang J, Jiang L, Liu J, Pinello L, Yuan GC, Shou C (2017). Gene regulatory pattern analysis reveals essential role of core transcriptional factors’ activation in triple-negative breast cancer. Oncotarget.

