# Correction to: AKT-mediated phosphorylation of Sox9 induces Sox10 transcription in a murine model of HER2-positive breast cancer

**DOI:** 10.1186/s13058-021-01440-9

**Published:** 2021-05-26

**Authors:** Khalid N. Al-Zahrani, John Abou-Hamad, Julia Pascoal, Cédrik Labrèche, Brennan Garland, Luc A. Sabourin

**Affiliations:** 1 Centre for Cancer Therapeutics, Ottawa Hospital Research Institute, 501 Smyth Road, Ottawa, ON K1H 8L6 Canada; 2 Department of Cellular and Molecular Medicine, University of Ottawa, Ottawa, ON K1H 8M5 Canada

**Correction to: Breast Cancer Res 23, 55 (2021)**

**https://doi.org/10.1186/s13058-021-01435-6**

After publication of the original article [1], the authors identified an error in Figure 2. The correct figure is given below.

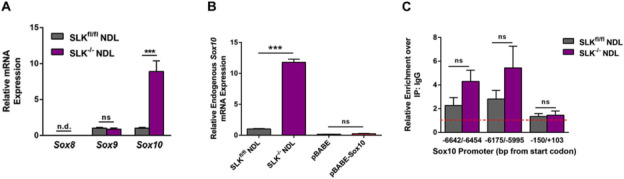



The original article has been corrected.
