# Supplementary figures and images for: AKT-mediated phosphorylation of Sox9 induces Sox10 transcription in a murine model of HER2-positive breast cancer
Source: Breast Cancer Res. 2021 May 13;23:55. doi: 10.1186/s13058-021-01435-6 (PMC8120776; doi:10.1186/s13058-021-01435-6)

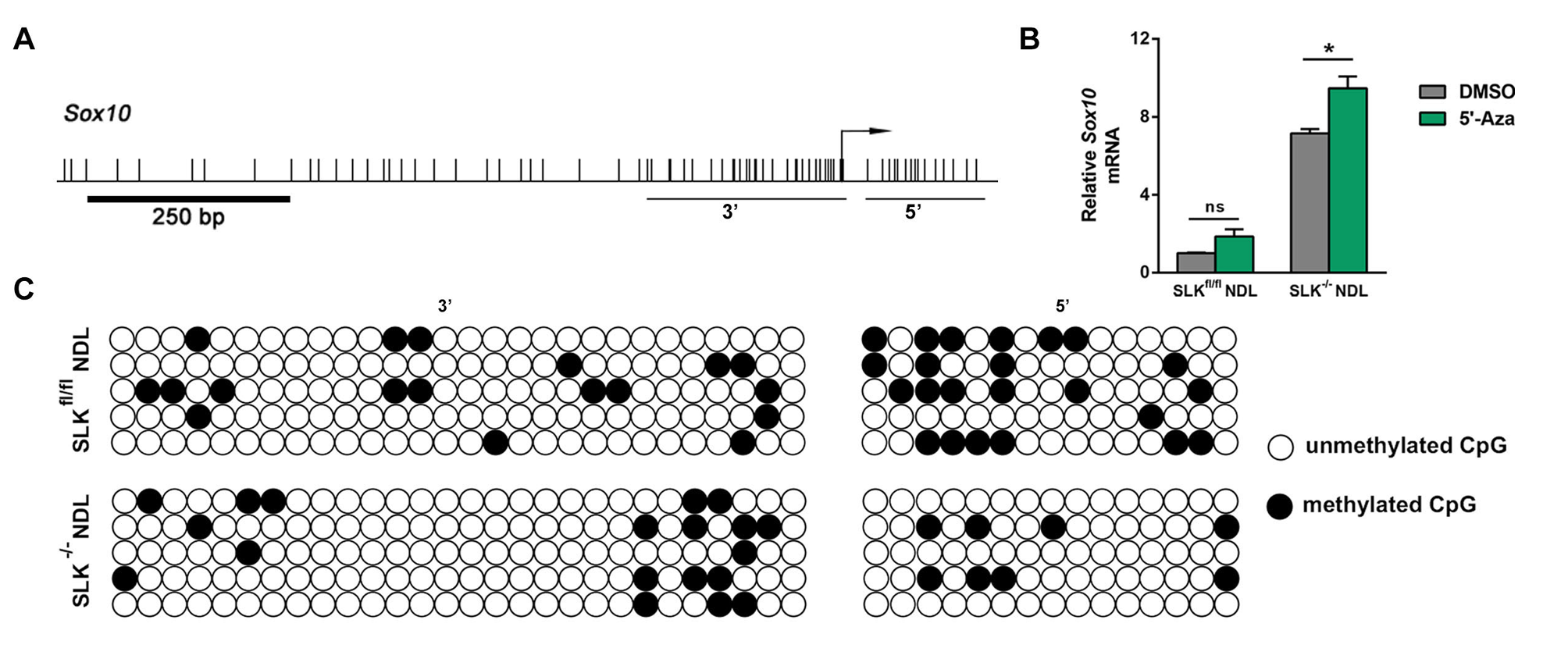

Supplement: Supplementary file 1 — Additional file 1: Supplementary Figure 1. Induction of Sox10 following Slk deletion is not due to promoter demethylation. A, Schematic representation of the murine Sox10 gene. The transcriptional start site is indicated by the forward arrow. The location of all CG dinucleotides are represented as individual vertical lines. Methprimer CpG prediction software was used to identify potential CpG islands and are shown as the underlined regions. B, SLKfl/fl and SLK-/- NDL cells were treated with 5-aza-2’-deoxycytidine (5’-Aza) for five days. The levels of Sox10 transcript was assessed by qPCR analysis. C, Bisulfite sequencing of genomic DNA from SLKfl/fl and SLK-/- NDL cells was performed. Five independent clones from each cell line was sequenced. A representative plot of unmethylated (open) and methylated (filled) CpG repeats from two putative CpG islands identified in (A) is presented. ns: no statistical difference, * p < 0.05. [file 13058_2021_1435_MOESM1_ESM.jpg]

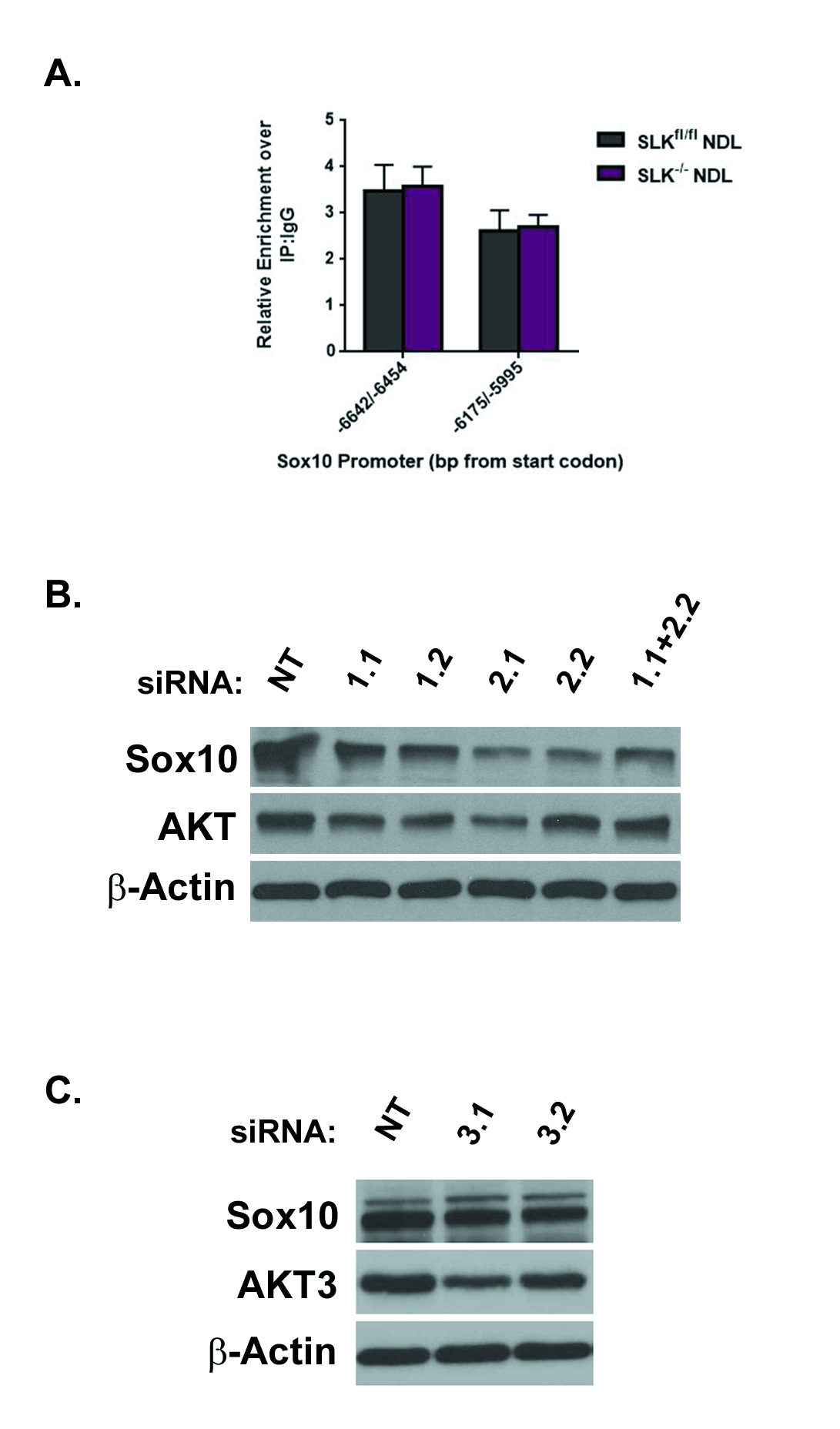

Supplement: Supplementary file 2 — Additional file 2: Supplementary Figure 2. Sox10 induction is preferentially mediated by AKT2. (A) Chromatin immunoprecipitation (ChIP) was performed on SLKfl/fl and SLK-/- NDL cells to assess K27 Acetylated histone H3 binding to the Sox10 enhancers. Following ChIP, qPCR analysis was performed across two putative SoxE binding sites within the -6904/-5995 fragment of the Sox10 promoter. qRT-PCR data was normalized to an IgG ChIP or a negative control element within exon one (-150/+103) as in Fig. 2. No statistical differences were observed between the cell lines. N=3. (B) Knock down of AKT1 or AKT2 results in Sox10 downregulation. AKT1 knockdowns were ~50% at best with two independent siRNAs. AKT2 knockdowns with two siRNAs ranged from 50-70% with a marked downregulation (80-90%) of Sox10. (C) AKT3 knockdowns from 40-60% did not show any appreciable reduction in Sox10 levels. Underexposed blots were subjected to ImageJ densitometry and normalized to β-actin and NT controls. NT= non targeting siRNA control. [file 13058_2021_1435_MOESM2_ESM.jpg]
