# Supplementary material for: AKT-mediated phosphorylation of Sox9 induces Sox10 transcription in a murine model of HER2-positive breast cancer
Source: Breast Cancer Res. 2021 May 13;23:55. doi: 10.1186/s13058-021-01435-6 (PMC8120776; doi:10.1186/s13058-021-01435-6)
Supplement: Supplementary file 3 — Additional file 3: Table S1. List of Primers. [file 13058_2021_1435_MOESM3_ESM.pdf]

Table S1. List of Primers

Primer	Assay	Forward Primer Sequence	Reverse Primer Sequence
Sox10 promoter -6904/-5995	Cloning	GCGGTACCCTCTTTCCTGTCCAA	CGCTCGAGAGCAGATCACATTGG
Sox10 promoter -4565/-3503	Cloning	GCGGTACCTCCCTGATACCACAT	CGCTCGAGATACCCTGGGGTGCT
Sox10 promoter -3484/-2495	Cloning	GCGGTACCTATTGACCTGAGGTG	CGCTCGAGTCCCTCCTCTTGGAG
Sox10 promoter -1895/+359	Cloning	CCGGTACCTAGGCTGTAGAGGTGAG	CCCTCGAGCATGTCGCTCCCGGCCG
pGEX-Sox9	Cloning	CAGGATCCAATCTCCTGGACCCCTTCATG	CACTCGAGTCAGGGTCTGGTGAGCTG
mSox9 S181A	Mutagenesis	GCCCCGGCGGAGGAAGGCGGTGAAGAACGGACAAGCGGAG	CTCCGCTTGTCCGTTCTTCACCGCCTTCCTCCGCCGGGGC
Sox10 distal promoter	Bisulfite Sequencing	ATTTATTTAGGGTTTGGTATGTGTA	CCACCTCTAATAAATCTTATTCCTC
Sox10 proximal promoter	Bisulfite Sequencing	ATTAGAGGTGGAGTTGAGTTTTGTG	ATTCCTTCTTAACCTTACCCAATTC
Sox8	qRT-PCR	TGGAGTCTGGTGCCTATGCCTGT	GCCGAGCACTGCATCAGCTTTGT
Sox9	qRT-PCR	CATCAAGACGGAGCAGCTGAG	ATGGTCAGCGTAGTCGTATTG
Sox10	qRT-PCR	AGGTTGCTGAACGAAAGTGAC	CCGAGGTTGGTACTTGTAGTCC
Sox10 3' UTR	qRT-PCR	CATCTAGAAGGGGCCCTGTCACCA	CAGGATCCCAGGTCAGGCCACCAAG
Sox10 promoter -6642	ChIP qRT-PCR	TGGGACCCGACGGCGGCGGCGGTG	CTTGCTCTTGCTGGCACCGTTGAC
Sox10 promoter -6175	ChIP qRT-PCR	CCCTCGCTCTGTCCAGATTAATG	AGCAGATCACATTGGTAGACATGTAG
Sox10 promoter -150	ChIP qRT-PCR	ACAGAGATGAGCCATGTGAAAATAAG	AGCTGTCAGCAGCCTGGGCAAGGG
