# Supplementary material for: AKT-mediated phosphorylation of Sox9 induces Sox10 transcription in a murine model of HER2-positive breast cancer
Source: Breast Cancer Res. 2021 May 13;23:55. doi: 10.1186/s13058-021-01435-6 (PMC8120776; doi:10.1186/s13058-021-01435-6)
Supplement: Supplementary file 4 — Additional file 4: Table S2. Antibodies. [file 13058_2021_1435_MOESM4_ESM.pdf]

Supplemental Table 2: Antibodies

Antibody	Company	Catalog Number	Western Blot	IHC-P
anti-SLK	custom made	-	1:10000	1:500
anti-β-actin	Sigma	A5316	1:20000	-
anti-Sox10	Novus	NBP2-44474	-	1:400
anti-Sox10	Cell Signaling Technology	89356	1:1000	-
anti-Sox9	Novus	NBP1-8555	1:2000	-
anti-pSox9 S181	Abcam	ab59252	1:500	1:100
anti-pAKT S473	Cell Signaling Technology	4060	1:1000	-
anti-AKT	Cell Signaling Technology	9272	1:2000	-
anti-CREB	Cell Signaling Technology	9197	1:1000	-
anti-FoxA1	Cell Signaling Technology	53528	1:1000	-
anti-FoxO1	Cell Signaling Technology	2880	1:1000	-
anti-FoxO3A	Cell Signaling Technology	12829	1:1000	-
anti-Lamin A/C	Cell Signaling Technology	4744	1:2000	-
anti-GAPDH	Cell Signaling Technology	2118	1:2000	-
anti-K27 acetyl-H3	Abcam	4729	-	-
