# Supplementary material for: AKT-mediated phosphorylation of Sox9 induces Sox10 transcription in a murine model of HER2-positive breast cancer
Source: Breast Cancer Res. 2021 May 13;23:55. doi: 10.1186/s13058-021-01435-6 (PMC8120776; doi:10.1186/s13058-021-01435-6)
Supplement: Supplementary file 5 — Additional file 5: Table S3. Akt siRNAs (Sigma). [file 13058_2021_1435_MOESM5_ESM.pdf]

Supplemental Table 3: Akt siRNAs (Sigma)

**siRNA**	**Sequence**
Non Targeting	CAGUCGCGUUUGCGACUGG
AKT1.1	AACGAUGGCACCUUUAUUGGC
AKT1.2	AACCAGGACCACGAGAAGCUG
AKT2.1	AAACUCCUCGGCAAGGGCACC
AKT2.2	AAGAGUGGAUGCGGGCUAUCC
AKT3.1	AAGGAUGAAGUGGCACACACU
AKT3.2	AAGAGGGUUGGGUUCAGAAGA
